# Factors Influencing Contrast Sensitivity Function in Eyes with Mild Cataract

**DOI:** 10.3390/jcm10071506

**Published:** 2021-04-04

**Authors:** Kazutaka Kamiya, Fusako Fujimura, Takushi Kawamorita, Wakako Ando, Yoshihiko Iida, Nobuyuki Shoji

**Affiliations:** 1 Visual Physiology, School of Allied Health Sciences, Kitasato University, Kanagawa 2520373, Japan; f-fujimu@kitasato-u.ac.jp (F.F.); kawa2008@kitasato-u.ac.jp (T.K.); 2 Department of Ophthalmology, School of Medicine, Kitasato University, Kanagawa 2520374, Japan; wakako@kitasato-u.ac.jp (W.A.); sparkle.i.4415@gmail.com (Y.I.); nshoji@kitasato-u.ac.jp (N.S.)

**Keywords:** contrast sensitivity, cataract, AULCSF, visual acuity, intraocular scattering, higher-order aberrations

## Abstract

This study was aimed to evaluate the relationship between the area under the log contrast sensitivity function (AULCSF) and several optical factors in eyes suffering mild cataract. We enrolled 71 eyes of 71 patients (mean age, 71.4 ± 10.7 (standard deviation) years) with cataract formation who were under surgical consultation. We determined the area under the log contrast sensitivity function (AULCSF) using a contrast sensitivity unit (VCTS-6500, Vistech). We utilized single and multiple regression analyses to investigate the relevant factors in such eyes. The mean AULSCF was 1.06 ± 0.16 (0.62 to 1.38). Explanatory variables relevant to the AULCSF were, in order of influence, logMAR best spectacle-corrected visual acuity (BSCVA) (*p* < 0.001, partial regression coefficient B = −0.372), and log(s) (*p* = 0.023, B = −0.032) (adjusted R^2^ = 0.402). We found no significant association with other variables such as age, gender, uncorrected visual acuity, nuclear sclerosis grade, or ocular HOAs. Eyes with better BSCVA and lower log(s) are more susceptible to show higher AULCSF, even in mild cataract subjects. It is indicated that both visual acuity and intraocular forward scattering play a role in the CS function in such eyes.

## 1. Introduction

Cataract still remains a major cause of visual impairment worldwide [1]. The prevalence rate of cataract increases with age, according to population-based studies on lens opacities [2,3]. Cataract has a greater impact on the quality of life of older adults, including increased difficulties in daily activities, compared with other common age-related conditions [4,5]. Cataract importantly increases scattered light, when light passes through the eye media, generating a veil of straylight over the retina that degrades vision, a phenomenon called straylight. This veiling luminance over the retina affects the retinal image quality, diminishing contrast and increasing the sensitivity to glare. Actually, it has been demonstrated that the amount of scattered light was objectively assessed by the double-pass instrument, as an objective scatter index (OSI) in cataract patients [6], and that this index can be used for cataract classification [7]. The comparison compensation method has been successfully applied in order to subjectively assess intraocular straylight by the logarithmic straylight value (log(s)) [8,9,10]. It has been shown that visual acuity and straylight are rather independent aspects of the overall quality of vision in cataract patients [11]. Cataracts have been reported to notably influence driving performance in older subjects, and that the OSI has high predictive power when it comes to simulated driving performance in older drivers [12]. Likewise, straylight has been shown to be the best parameter for predicting simulated driving performance in older drivers [13]. It has also been known that visual functions apart from visual acuity may be more associated with visual complaints that impact the quality of life.

Since conventional visual acuity testing may not be suitable for the assessment of detailed visual quality [14], contrast sensitivity (CS) testing will be clinically helpful for this evaluation, especially in eyes with mild cataract. Adamsons et al. stated that preoperative measurement of contrast sensitivity can help determine who with early cataract with mild impairment in visual acuity is most likely to report subjective improvement in vision [15]. Superstein et al. showed that spatial contrast sensitivity testing provided an objective assessment of patients who had good visual acuity yet also had functional complaints [16], and that should be considered as adjuncts to visual acuity testing in evaluating certain cataract patients [17]. The deterioration in CS function is caused not only by cataract formation itself, but also by the aging process and its consequent effect on visual processing and on the retina. We previously reported that intraocular forward scattering plays a more vital role in CS function than higher-order aberrations (HOAs) in myopic subjects [18]. However, the effect of light scatter and HOAs on CS function has not been fully elucidated in eyes having mild cataract. It may provide basic insights on understanding detailed visual performance in mild cataract patients. The goal of the current study is twofold; to quantitatively determine CS function in eyes with mild cataract, and to assess the background factors affecting CS function using single and multiple regression analyses in such eyes.

## 2. Materials and Methods

### 2.1. Study Population

We registered with the University Hospital Medical Information Network Clinical Trial Registry (000034854). Seventy-one eyes of 71 consecutive subjects (mean age ± standard deviation; 71.4 ± 10.7 years, 34 men and 37 women), who completed optical examinations for cataract surgery consultation, and who had no other ocular diseases, except for mild cataract, were enrolled in the current study. Only subjects in whom we could reproducibly quantify all optical parameters using the straylight meter, as well as the Hartmann–Shack aberrometry, were defined as mild cataract in this study. We randomly selected only one eye per subject for statistical analysis, when a bilateral cataract occurred. This retrospective study was approved by the Institutional Review Board at Kitasato University Hospital (B16-67), and followed the tenets of the Declaration of Helsinki. Our Institutional Review Board waived the requirement for informed consent for this retrospective review.

### 2.2. Assessment of Contrast Sensitivity Function

We measured the CS function using a contrast sensitivity unit (VCTS-6500, Vistech) under photopic conditions (500 lux). We conducted this test with the best spectacle correction at a distance of 2.5 m. We determined the area under the log contrast sensitivity function (AULCSF) by the CS data, as described previously [19]. Briefly, we plotted the log of CS as a function of log spatial frequency and fitted third-order polynomials to the data. We integrated the fitted function between the fixed limits of log spatial frequencies of 0.18 (corresponding to 1.5 cycles/degree) to 1.26 (corresponding to 18 cycles/degree), and determined the obtained value as the AULCSF.

### 2.3. Assessment of Visual Acuity, Nuclear Sclerosis and Cataract Type

We performed visual acuity measurement using a Snellen chart at 5 m, with and without spectacle correction. Two cataract specialists assessed the grade of nuclear sclerosis of the crystalline lens according to the Emery-Little classification, and the cataract type was divided into three subgroups (nuclear sclerosis, cortical, and posterior subcapsular cataract subgroups), based on slit-lamp biomicroscopy after mydriasis. We defined as cases those subjects who presented with an advanced form of 1 of the 3 types of cataract, regardless of the concomitant presence of the remaining 2 types of cataract. In addition, we investigated the relationship of the AULCSF with the logarithm of the minimal angle of resolution (logMAR) of best spectacle-corrected visual acuity (BSCVA) and log(s) in early cataract eyes with logMAR BSCVA of 0.05 or better.

### 2.4. Assessment of Intraocular Forward Scattering and Higher-Order Aberrations

We measured the retinal straylight, as a measure of subjective forward scattering, using the C-Quant straylight meter (Oculus Optikgeräte, GmbH, Wetzlar, Germany). Briefly, a test field that consists of a dark circle divided into two semicircles and is surrounded by a ring-shaped flickering light. A counter-phase compensation light is presented in one of the semicircles, reducing the flicker perception on that side. The subjects are instructed to select which semicircle is flickering more intensely. We repeated this process 3 times with different levels of compensation light, resulting in a logarithmic straylight value (log(s)) [8,9,10]. We used the measurement only when the estimated standard deviation was <0.08 and the quality factor for psychometric sampling was >1.00 [9].

We determined ocular HOAs for a 4-mm pupil after mydriasis using the Hartmann-Shack aberrometry (KR-1W, Topcon, Tokyo, Japan). We separately calculated the root mean square of the 3rd- and 4th-order coefficients.

### 2.5. Statistical Analysis

We used commercially-available statistical software (Bellcurve for Excel, Social Survey Research Information Co, Ltd., Tokyo, Japan) for statistical analyses. We conducted stepwise multiple regression analysis to assess the relationship of the CS function with several parameters. We utilized the AULCSF as the dependent variable, and age, gender, logMAR of uncorrected visual acuity (UCVA) and BSCVA, nuclear sclerosis grade, log(s), ocular 3rd-order aberrations, and ocular 4th-order aberrations as the explanatory variables. We also conducted Spearman’s rank correlation test to evaluate the relationships between the AULCSF and other variables. We applied a one-way analysis of variance for the analysis of the AULCSF among the 3 cataract subgroups. We described the results as mean ± standard deviation, and deemed a *p*-value < 0.05 statistically significant.

## 3. Results

**Figure 1:**
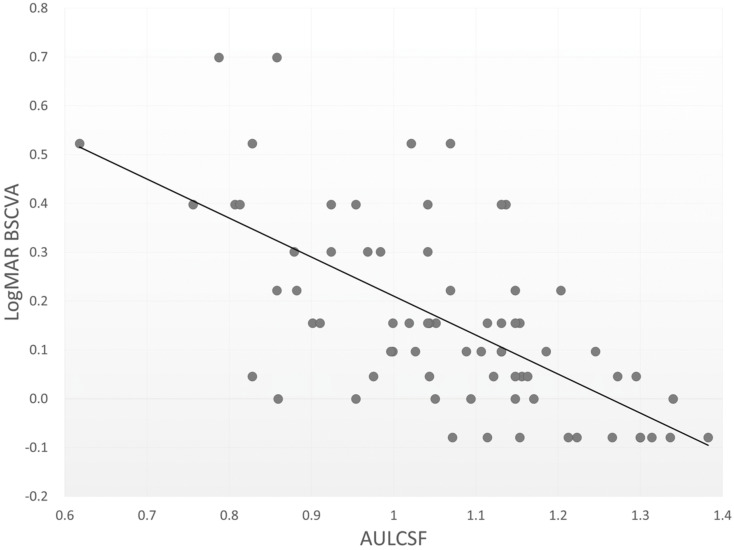
A scatterplot between the AULSCF and logMAR best spectacle-corrected visual acuity (Spearman correlation coefficient r = −0.640, *p* < 0.001).

**Figure 2:**
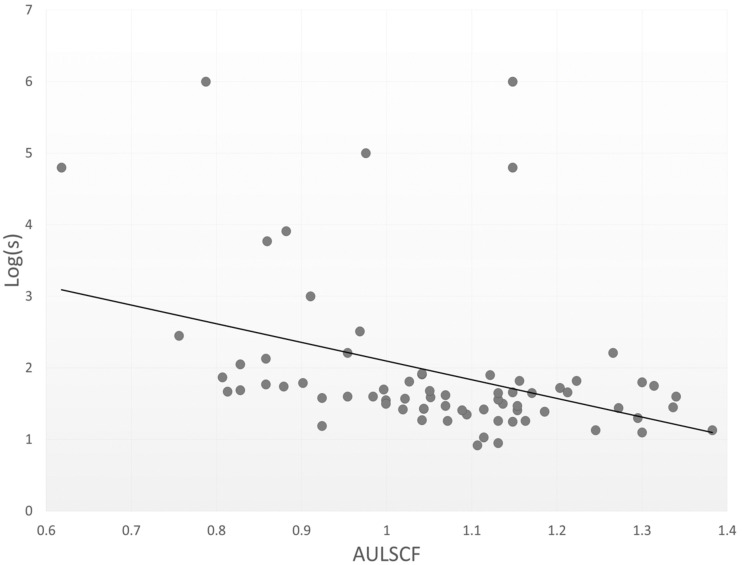
A scatterplot between the AULSCF and log(s) (Spearman correlation coefficient r = −0.427, *p* < 0.001).

Table 1 shows the patient demographics in the present study. The mean AULSCF was 1.06 ± 0.16 (range, 0.62 to 1.38). The AULCSF was 1.07 ± 0.11, 1.08 ± 0.16, and 1.01 ± 0.20, in the nuclear sclerosis, cortical, and posterior subcapsular cataract subgroups. We found no significant differences in the AULCSF among the three subgroups (analysis of variance, *p* = 0.391). Table 2 summarizes the results of multiple regression analysis. The relevant explanatory variables were logMAR BSCVA (*p* < 0.001, partial regression coefficient B = −0.372) and log(s) (*p* = 0.023, B = −0.032) (adjusted R^2^ = 0.402). The equation was described as follows: AULCSF = (−0.372 × logMAR BSCVA) + (−0.032 × log(s)) + 1.385. There were no significant associations with other explanatory variables such as age, gender, UCVA, nuclear sclerosis grade, ocular 3rd-order HOAs, or ocular 4th-order HOAs. The standardized partial regression coefficient was determined in order to investigate the level of each variable’s influence. The most relevant variable was logMAR BSCVA, followed by the log(s). Table 2 shows similar results by single regression analysis. Figure 1 and Figure 2 show significant associations between the AULCSF and logMAR BSCVA (r = −0.640, *p* < 0.001), and those between the AULCSF and the log(s) (r = −0.427, *p* < 0.001), respectively. With better BSCVA, lower log(s), or both, the AULSCF became significantly higher in eyes having mild cataract. On the other hand, we found no significant correlations of the AULCSF with ocular 3rd-order aberrations (r = −0.144, *p* = 0.264), or 4th-order aberrations (r = −0.167, *p* = 0.194). For subgroup analysis in 26 early cataract eyes with logMAR BSCVA of 0.05 or better, we also found significant correlations between the AULCSF and logMAR BSCVA (r = −0.388, *p* = 0.049), and those between the AULCSF and the log(s) (r = −0.405, *p* = 0.040), but no significant correlations between the AULCSF and 3rd-order aberrations (r = −0.249, *p* = 0.220), or those between the AULCSF and 4th-order aberrations (r = −0.128, *p* = 0.532).

## 4. Discussion

In the current study, our findings showed that both BSCVA and log(s) were significantly correlated with the CS function in eyes with mild cataract, although some of the variance has remained unanswered, as confirmed by the moderate R^2^ value (0.402). Since CS can be affected by multiple factors, such as retina and brain processing [20,21], it is reasonable that the CS function cannot be totally clarified by the optics. To the best of our knowledge, this is the first study to determine the detailed clinical factors affecting the CS function by single and multiple regression analyses in mild cataract subjects.

With regard to visual acuity and CS function for cataract, Adamsons et al. described that the CS scores were lower for patients having mild lens opacities than for patients having clear lenses at high spatial frequencies, suggesting that decreased visual function for patients with early cataracts whose visual acuity is only minimally impaired [22]. Fujikado et al. reported that the AULCSF was moderately associated with the HOAs as well as with intraocular scattering in eyes having cataract [23]. Shandiz et al. found a significant loss of CS at all frequencies with increasing cataract severity, indicating that the AULCSF may provide additional information compared with standard visual acuity tests in patients with early cataracts [24]. Visual acuity encompasses a narrow central visual angle (0.02 degrees), whereas CS encompasses an angle of approximately 0.30 degrees. It is understandable that BSCVA was significantly associated with the AULSCF in the present study. It is suggested that BSCVA is one of the most relevant factors influencing the CS function for clinical use, even in eyes having mild cataract.

With regard to log(s) and CS function for cataract, van den Berg et al. and van der Meulen et al. demonstrated that visual acuity was not strongly correlated with straylight, indicating that each measurement shows different aspects of quality of vision [11,25]. Palomo-Álvarez et al. stated that the mean straylight (1.38 ± 0.24) in the cataract group was significantly worse than that (1.17 ± 0.11) in the control group [26]. Their findings of log(s) in cataract patients were slightly lower than our findings, presumably because of the differences in patient age (67.96 ± 7.11 years vs. 71.4 ± 10.7 years), cataract type, and cataract grade. Paz Filgueira et al. showed that straylight meter measurements demonstrate the loss of CS resulting from nuclear and posterior subcapsular opacities [27]. Martínez-Roda et al. found significant associations of the grading according to the lens opacities classification system III [28] with log(s) and OSI, although they were slightly stronger with OSI for all cataract types [29]. These previous and our current findings indicate that the increase in intraocular forward scattering caused by the changes in the transparency of the crystalline lens, contributes to the loss of CS function.

With regard to HOAs and CS function for cataract, we found no significant associations of the AULCSF with ocular 3rd-order or 4th-order aberrations in mild cataract population in the current study. Kuroda et al. mentioned that both light scattering and optical aberration of the lens leads to the loss of CS in mild cataract [30]. Fujikado et al. also found a significant correlation between the AULCSF and HOAs in cataract population [23]. The differences in the sample size, the methodology of the measurements, the distribution of patient age, cataract severity, and other background factors, may explain this discrepancy between the previous and current findings.

We have several limitations to this study. Firstly, it was performed in a retrospective fashion, and there was no control group without cataract. Considering that straylight was subjectively assessed using the compensation comparison method, a randomized, controlled study with a control group may provide further information for confirming our findings. Secondly, we only included mild cataract subjects in whom we could reliably quantify all optical metrics with these devices. Accordingly, the study population might be biased, since severe cases that were not measurable for these metrics, including dense and mature cataracts, were excluded from the present study. Thirdly, we evaluated the CS function only under photopic conditions. Although the CS function under mesopic and scotopic conditions is likely to be somewhat related to that under photopic conditions, a further study under such conditions would be ideal to confirm our findings. Fourthly, our optical findings might be influenced by other functions, such as cognitive function or motor function in these older patients, especially in the case of the C-Quant testing, although we confirmed that all participants had no history of cognitive or motor impairment.

## 5. Conclusions

In summary, our findings demonstrated that eyes with better BSCVA and eyes with lower log(s) showed higher AULCSF in eyes having mild cataract, although the most variance remained unclear. Based on our results, both visual acuity and intraocular forward scattering play some role in predicting the CS function in mild cataract subjects. Further research in a large cohort of cataract patients with various stages will be necessary to confirm the authenticity of these results.

## Figures and Tables

**Table 1:** Demographic and visual functional data of the study population.

Patient Demographics and Visual Function	
Age (years)	71.4 ± 10.7 years (95% CI, 50.5 to 92.3 years)
Gender (Male:Female)	34:37
LogMAR UCVA	0.67 ± 0.49 (95% CI, −0.29 to 1.62)
LogMAR BSCVA	0.16 ± 0.19 (95% CI, −0.22 to 0.54)
Sphere (D)	−1.10 ± 4.18 D (95% CI, −9.28 to 7.09 D)
Cylinder (D)	1.05 ± 0.90 D (95% CI, −0.71 to 2.80 D)
Grade of nuclear sclerosis	2.08 ± 0.19 (95% CI, 1.16 to 3.02)
Log(s)	1.93 ± 1.08 (95% CI, −0.18 to 4.05)
Ocular 3rd-order aberrations (µm)	0.21 ± 0.12 µm (95% CI, −0.03 to 0.45 µm)
Ocular 4th-order aberrations (µm)	0.13 ± 0.06 µm (95% CI, 0.01 to 0.25 µm)
AULCSF	1.06 ± 0.16 (95% CI, 0.75 to 1.37)

CI = confidence interval, logMAR = logarithm of the minimal angle of resolution, UCVA = uncorrected visual acuity, BSCVA = best spectacle-corrected visual acuity, D = diopter, AULSCF = area under the log contrast sensitivity function.

**Table 2:** Results of correlation analysis and stepwise multiple regression analysis to select variables relevant to the area under the log contrast sensitivity function (AULCSF) in eyes with mild cataract.

Variables	Spearman Correlation Coefficient	*p*-Value	Partial Regression Coefficient	Standardized Partial Regression Coefficient	*p*-Value
Log(s)	−0.427	<0.001	−0.032	−0.241	0.023
LogMAR BSCVA	−0.640	<0.001	−0.372	−0.467	<0.001
Age (years)	−0.149	0.213	not included		-
Gender (male = 0, female = 1)	0.096	0.428	not included		-
LogMAR UCVA	−0.150	0.211	not included		-
Grade of nuclear sclerosis	−0.128	0.287	not included		-
Ocular 3rd-order aberrations (µm)	−0.144	0.264	not included		-
Ocular 4th-order aberrations (µm)	−0.167	0.194	not included		-
			1.385	Constant	

LogMAR = logarithm of the minimal angle of resolution, BSCVA = best spectacle-corrected visual acuity, UCVA = uncorrected visual acuity.

## Data Availability

The data that support the findings of this study are available from the corresponding author, K.K., upon reasonable request.

## Abbreviations

CS: contrast sensitivity; HOAs: higher-order aberrations; AULCSF: area under the log contrast sensitivity function; logMAR: logarithm of the minimal angle of resolution; log(s): logarithmic straylight; UCVA: uncorrected visual acuity; BSCVA: best spectacle-corrected visual acuity.
